# Inhibition of Autophagy Potentiates Atorvastatin-Induced Apoptotic Cell Death in Human Bladder Cancer Cells *in Vitro*

**DOI:** 10.3390/ijms15058106

**Published:** 2014-05-08

**Authors:** Minyong Kang, Chang Wook Jeong, Ja Hyeon Ku, Cheol Kwak, Hyeon Hoe Kim

**Affiliations:** 1 Graduate School of Medical Science and Engineering (GSMSE), Korea Advanced Institute of Science and Technology (KAIST), 291 Daehak-ro, Yuseong-gu, Daejeon 305-701, Korea; E-Mail: dr.minyong.kang@gmail.com; 2 Department of Urology, Seoul National University Hospital, 101 Daehak-ro, Jongno-gu, Seoul 110-744, Korea; E-Mails: drboss@snuh.org (C.W.J.); kuuro70@snu.ac.kr (J.H.K.); mdrafael@snu.ac.kr (C.K.)

**Keywords:** urinary bladder neoplasms, atorvastatin, apoptosis, autophagy

## Abstract

Statins are cholesterol reduction agents that exhibit anti-cancer activity in several human cancers. Because autophagy is a crucial survival mechanism for cancer cells under stress conditions, cooperative inhibition of autophagy acts synergistically with other anti-cancer drugs. Thus, this study investigates whether combined treatment of atorvastatin and autophagy inhibitors results in enhancing the cytotoxic effects of atorvastatin, upon human bladder cancer cells, T24 and J82, *in vitro*. To measure cell viability, we performed the EZ-Cytox cell viability assay. We examined apoptosis by flow cytometry using annexin-V/propidium iodide (PI and western blot using procaspase-3 and poly (ADP-ribose) polymerase (PARP) antibodies. To examine autophagy activation, we evaluated the co-localization of LC3 and LysoTracker by immunocytochemistry, as well as the expression of LC3 and p62/sequestosome-1 (SQSTM1) by western blot. In addition, we assessed the survival and proliferation of T24 and J82 cells by a clonogenic assay. We found that atorvastatin reduced the cell viability of T24 and J82 cells via apoptotic cell death and induced autophagy activation, shown by the co-localization of LC3 and LysoTracker. Moreover, pharmacologic inhibition of autophagy significantly enhanced atorvastatin-induced apoptosis in T24 and J82 cells. In sum, inhibition of autophagy potentiates atorvastatin-induced apoptotic cell death in human bladder cancer cells *in vitro*, providing a potential therapeutic approach to treat bladder cancer.

## 1. Introduction

Urinary bladder cancer is the ninth most common cancer in the world, posing a crucial public health problem because of its high aggressiveness and poor prognosis [1]. In new bladder cancer cases, 70% to 80% are non-muscle invasive tumors that can be managed by combined therapy with transurethral resection and intravesical chemotherapy [2]. However, more than 50% of superficial bladder cancers will recur, and eventually 10% to 20% of recurrent tumors give rise to muscle invasive tumors [3]. Radical cystectomy with pelvic lymphadenectomy is the current gold standard treatment for muscle-invasive bladder cancer [4]. Given the high recurrence rates even after surgical resection, multimodal therapy consisting of surgical approaches combined with radiotherapy and chemotherapy is typically considered for the patients with muscle-invasive bladder cancer [5]. Nonetheless, such a therapeutic strategy still has unfavorable clinical outcomes, and there is growing interest in alternative therapeutic approaches to manage bladder cancer.

Statins block cholesterol biosynthesis by specifically inhibiting 3-hydroxy-3-methylglutaryl coenzyme A (HMG-CoA) reductase. They are widely used for the reduction of cholesterol and cardiovascular disease risk [6]. Emerging evidence reveals that statins also have anti-cancer effects for several types of cancer [7]. These drugs demonstrate pleiotropic activity to induce cell cycle arrest, apoptosis, inhibition of metastasis and angiogenesis and reversion of multidrug resistance. For bladder cancer, clinical studies yielded conflicting results on the cancer prevention effects of statins in patients with bladder cancer [8–10]. Some investigations did not find any association of statin use with anti-cancer effects on bladder cancer, while others suggested that statins increase the risk of bladder cancer [10,11]. However, recent multivariable analysis showed that statin use was not related to oncologic outcomes, including recurrence rate and cancer-specific mortality, in muscle-invasive bladder cancer [12]. In addition, *in vitro* studies showed that statins exhibited anti-cancer effects in human bladder cancer cell lines [13,14]. Given that combined treatments of statins with chemotherapeutic agents showed promising pre-clinical outcomes for treating several types of cancer [15], further research is needed to clearly define the usefulness of statins in treating bladder cancer.

Autophagy is an evolutionarily conserved, intracellular catabolic mechanism that degrades long-lived organelles and protein aggregates by fusion with lysosomes [16]. Because autophagy activation is closely associated with various stress conditions, dysfunction of autophagy is linked to a number of human diseases, including cancer [17]. Therefore, autophagy has received great attention as a novel target of cancer therapy [18]. Autophagy is highly activated in the hypoxic, nutrient poor regions of tumor, because cancer cells utilize autophagy to tolerate environmental stress [19]. Downregulating the expression of proteins associated with autophagy-lysosomal pathway attenuates the survival and growth of cancer cells in an energy and nutrient deprivation state [20]. Interestingly, statins induce autophagy activation via the adenosine monophosphate-activated protein kinase (AMPK)-mammalian target of rapamycin (mTOR) signaling pathway in cancer cells [21]. Because autophagy activation can promote the survival of cancer cells [22], statin-induced autophagy activation might be a mechanism to reduce the anti-cancer effect of statins. To our knowledge, there are no investigations of the relationship between statin use, autophagy activity and anti-cancer effects in bladder cancer cells.

In this study, we examined the effects of atorvastatin, a statin drug, on cytotoxicity and autophagy activation in human bladder cancer cells and evaluated the impact of autophagy inhibition on the effects of atorvastatin. We found that treatment with atorvastatin reduced cell viability by inducing apoptosis and triggered autophagy activation in T24 and J82 human bladder cancer cells. Furthermore, pharmacologic inhibition of autophagy significantly enhanced atorvastatin-induced cytotoxicity by promoting apoptotic cell death, providing the biological basis of a novel approach to treat bladder cancer.

## 2. Results and Discussion

### 2.1. Results

#### 2.1.1. Cytotoxic Effects of Atorvastatin against T24 Human Bladder Cancer Cells

**Figure 1.**
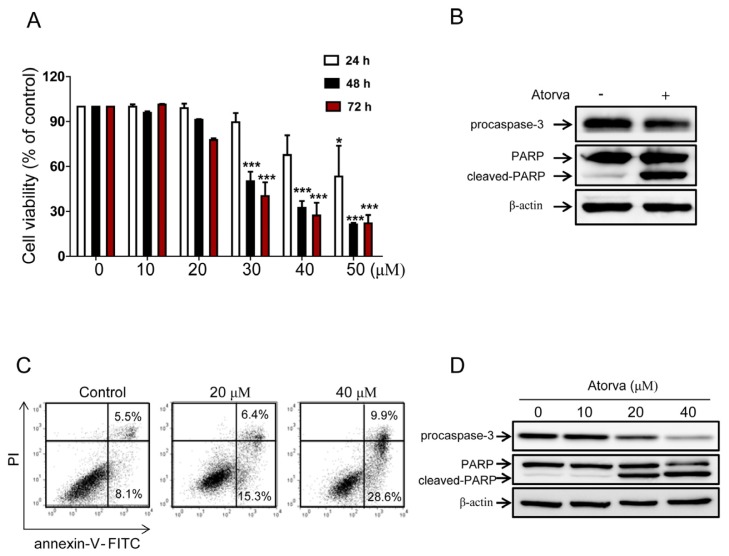

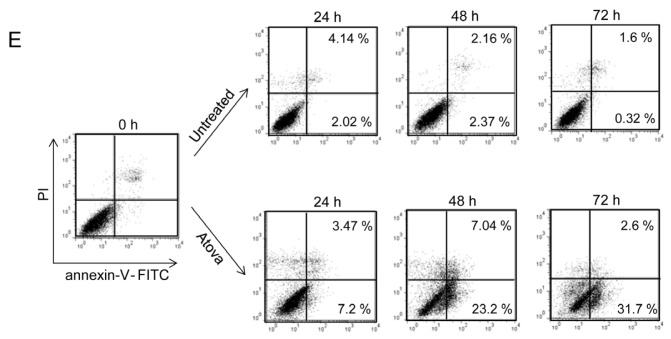
Cytotoxic effects of atorvastatin against T24 human bladder cancer cells. (**A**) The cell viability assay to examine the cytotoxic effects of atorvastatin in T24 cells. Differing concentrations of atorvastatin (zero, 10, 20, 30, 40 and 50 μM) were applied over three different durations (24, 48 and 72 h). The values of cell viability are represented by the mean percent of control ± SEM (*n* = 3, * *p* < 0.05, *** *p* < 0.001); (**B**) Western blot analysis of apoptotic markers, procaspase-3, total PARP and cleaved-PARP, in untreated (control) and atorvastatin (30 μM) treated T24 cells; (**C**) Evaluation of apoptotic cell death after treatments with 20 and 40 μM of atorvastatin in T24 cells by flow cytometry analysis with Fluorescein isothiocyanate (FITC)-conjugated annexin-V and propidium iodide (PI) staining. Relative proportions of both early and late apoptosis are indicated in right lower and right upper quadrant, respectively in each treatment group; (**D**) Western blot analysis of apoptotic markers procaspase-3, total PARP and cleaved-PARP in T24 cells treated with various concentration of atorvastatin (zero, 10, 20 and 40 μM); (**E**) Flow cytometry analysis with FITC-conjugated annexin-V and PI staining to examine apoptotic cell death after treatments with atorvastatin (30 μM) in T24 cells over different durations (24, 48 and 72 h). Relative proportions of both early and late apoptosis are indicated in right lower and right upper quadrant, respectively in each treatment group.

In the cells treated for 24 h, only the 50 μM concentration of atorvastatin reduced cell viability remarkably compared to a control, whereas 30, 40 and 50 μM concentrations reduced cell viability significantly after 48 and 72 h of treatment (Figure 1A). These results show that atorvastatin can reduce the cell viability of bladder cancer cells in a dose- and time-dependent manner. To determine if the cytotoxic effects of atorvastatin act by causing apoptotic cell death, the expression levels of apoptosis related factors were assessed by western blot analysis. As shown in Figure 1B, cleaved Poly (ADP-ribose) polymerase (PARP) increased, whereas procaspase-3 decreased in atorvastatin treated cells. In addition, flow cytometry analysis with annexin-V/propidium iodide (PI) double staining showed that apoptotic cell death increased after treatment with 20 and 40 μM of atorvastatin in a dose-dependent manner (Figure 1C). Western blot analysis demonstrated that cleaved PARP increased, whereas total PARP and procaspase-3 decreased in a dose-dependent manner (Figure 1D). Furthermore, apoptotic cell death induced by atorvastatin increased in a time-dependent manner, shown in flow cytometry (Figure 1E). These results indicate that atorvastatin has cytotoxic effects via the induction of apoptotic cell death in T24 human bladder cancer cells.

#### 2.1.2. Autophagy Induction by Atorvastatin Treatment in T24 Human Bladder Cancer Cells

**Figure 2.**
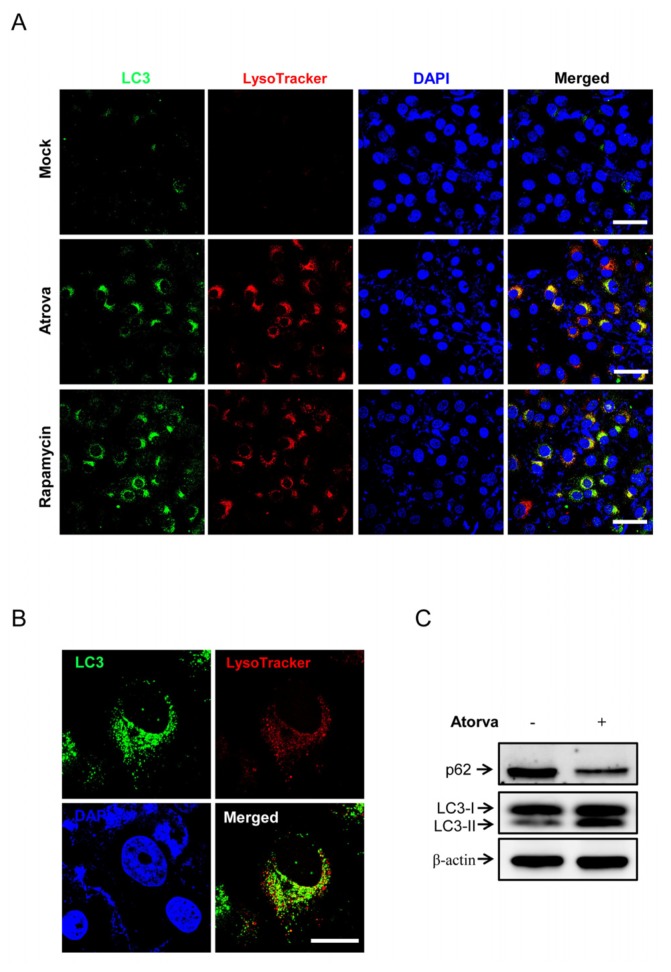
Autophagy induction by atorvastatin in T24 human bladder cancer cells. (**A**) Immunocytochemistry for the co-localization of LC3 puncta (green) and Lysotracker (red, demarcation for the lysosome) after atorvastatin treatments (30 μM) for 24 h in T24 cells. Rapamycin treated T24 cells were the positive control of autophagy induction. 4′,6-diamidino-2-phenylindole (DAPI) was used for nucleus staining. Scale bar = 100 μm; (**B**) High magnification view of immunocytochemistry for the co-localization LC3 puncta (green) and Lysotracker after atorvastatin treatments (30 μM) for 24 h in T24 cells at the single-cell level. Scale bar = 50 μm; (**C**) Western blot analysis of autophagosome formation markers p62/SQSTM1, LC3-I and LC3-II in untreated (control) and atorvastatin (30 μM) treated T24 cells.

To determine whether atorvastatin activates autophagy in bladder cancer cells, the expression patterns of endogenous LC3 and p62/sequestosome 1 (SQSTM1) were evaluated after treatment with atorvastatin. Immunocytochemistry showed that punctuated LC3, a key marker of autophagosome formation, was markedly detected in T24 cells treated with 30 μM of atorvastatin for 24 h, not in untreated cells (Figure 2A). To further detect lysosomal localization of LC3 puncta, we evaluated the co-localization of endogenous LC3 and a lysosomal marker (Lysotracker). As shown in Figure 2A (middle panels), atorvastatin treatments induced the co-localization of LC3 puncta (green) and Lysotracker (red) in the cytoplasm of T24 cells. Rapamycin treated cells were the positive control of autophagy induction by showing the co-staining of LC3 puncta and the lysosome in the cytoplasm of T24 cells (Figure 2A, lower panels). LC3 puncta were also highly conspicuous after atorvastatin treatment in the lysosome co-staining when observed at the single-cell level with high magnification (Figure 2B). In western blot analysis, conversion of LC3-I into LC3-II increased, while the protein level of p62/SQSTM1 decreased, indicating autophagy activation, in T24 human bladder cancer cells treated with atorvastatin (Figure 2C). To examine whether the inhibition of mevalonate pathway was directly involved in the atorvastatin-induced autophagy, T24 cells were treated with mevalonate. We found that treatment with mevalonate did not affect LC3-II accumulation triggered by atorvastatin (Figure S1). These results suggest that atorvastatin activates autophagosome formation in T24 human bladder cancer cells.

#### 2.1.3. Autophagy Inhibition Enhances Atorvastatin-Induced Apoptotic Cell Death in T24 Bladder Cancer Cells

**Figure 3.**
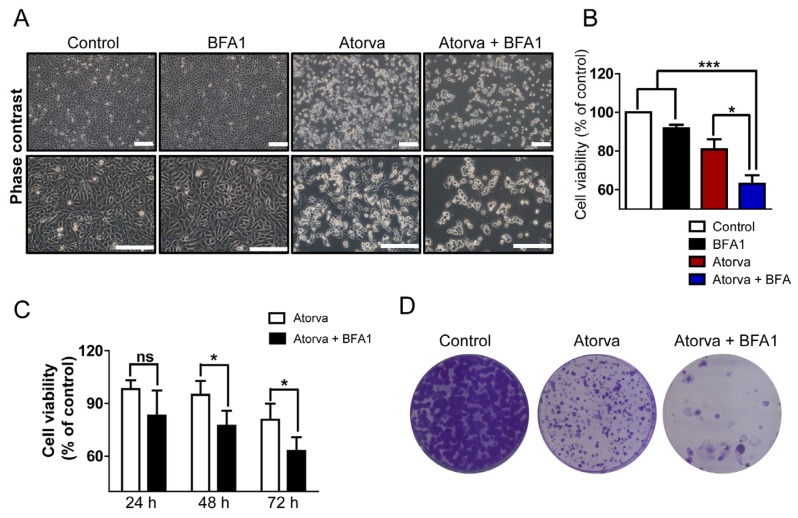

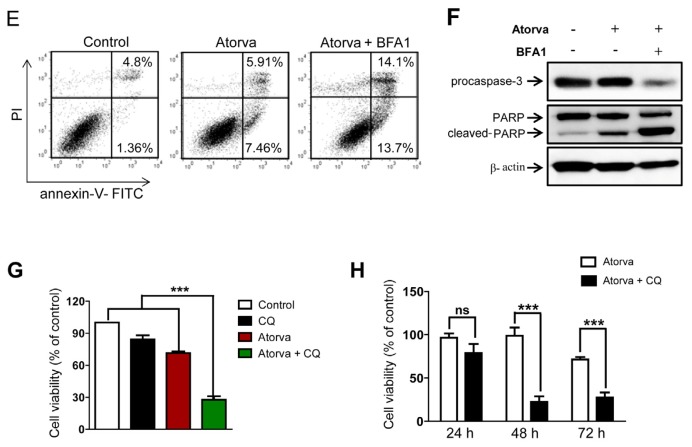
Autophagy inhibition enhances atorvastatin-induced apoptotic cell death in T24 bladder cancer cells. (**A**) Phase contrast images of untreated T24 cells (control), treated T24 cells with 20 μM of bafilomycin A1 (BFA1), 20 μM of atorvastatin (Atorva) and both these agents (Atorva + BFA1) for 48 h. Scale bar = 200 μm; (**B**) The cell viability assay to examine the cytotoxic effects of 20 μM of bafilomycin A1 (BFA1), 20 μM of atorvastatin (Atorva) and both these agents (Atorva + BFA1). The values of cell viability are represented by the mean percent of control ± SEM (*n* = 3, * *p* < 0.05, *** *p* <0.001); (**C**) The cell viability assay to compare the cytotoxic effects of 20 μM of atorvastatin and combined treatment with 20 μM of BFA1 and atorvastatin over three different durations (24, 48 and 72 h). Representative values of cell viability are represented by the mean percent of control ± SEM (*n* = 3, * *p* < 0.05; ns, not significant); (**D**) The clonogenic assay to compare *in vitro* cell survival potential after treatment with 20 μM of atorvastatin and combined treatment with 20 μM of atorvastatin and BFA1 for 12 days. The colony is defined as containing at least 50 individual cells. Photographs represent each experimental group stained with the clonogenic assay kit; (**E**) Flow cytometry analysis with FITC-conjugated annexin-V and PI staining to examine apoptotic cell death in T24 cells treated with 20 μM of atorvastatin and combined treatment with 20 μM of BFA1 and atorvastatin for 48 h. Relative proportions of both early and late apoptosis are indicated in right lower and right upper quadrant, respectively in each treatment group; (**F**) Western blot analysis of apoptotic markers procaspase-3, total PARP and cleaved-PARP in T24 cells treated with 20 μM of atorvastatin and both 20 μM of BFA1 and atorvastatin for 48 h; (**G**) The cell viability assay to examine the cytotoxic effects in T24 cells treated with 20 μM of chloroquine (CQ), 20 μM of atorvastatin and both these agents. The values of cell viability are represented by the mean percent of control ± SEM (*n* = 3, *** *p* < 0.001); (**H**) The cell viability assay to compare the cytotoxic effects in T24 cells treated with 20 μM of CQ, 20 μM of atorvastatin and both these agents over three different durations (24, 48 and 72 h). Representative values of cell viability are represented by the mean percent of control ± SEM (*n* = 3, *** *p* < 0.001; ns, not significant).

As shown in phase contrast images, a higher proportion of cells were damaged by treatments with both atorvastatin and autophagy inhibitor, bafilomycin A1 (BFA1), compared to either group treated with a single agent (Figure 3A). Cell viability was significantly reduced when treated with both agents compared to single agent treatment (Figure 3B). The enhanced cytotoxic effects of combined treatment with atorvastatin and BFA1 developed in a time-dependent manner (Figure 3C). As shown in Figure 3D, co-treatment with atorvastatin and BFA1 resulted in the prominent reduction of the clonogenic capacity of T24 cells compared to untreated and atorvastatin-only treated cells. Flow cytometry analysis showed that apoptotic cell death increased in both agents treating T24 cells (Figure 3E). Western blot analysis confirmed that combined treatment with atorvastatin and BFA1 increased the detection of cleaved-PARP, while decreasing total PARP and procaspase-3 (Figure 3F). Of note, treatment with BFA1, which blocks the lysosomal degradation of autophagosomes [23], resulted in prominent LC3-II accumulation regardless of whether or not the treatment with atorvastatin (Figure S2). To examine further if other autophagy inhibitor potentiate the effects of atorvastatin, chloroquine (CQ) was used as another autophagy inhibition method. Similar to BFA1, combined treatment with CQ significantly improved the cytotoxic effects of atorvastatin in a time-dependent manner (Figure 3G,H). In sum, combined treatment with atorvastatin and autophagy inhibitors improves the cytotoxic effects of atorvastatin in T24 human bladder cancer cells by promoting apoptotic cell death.

#### 2.1.4. Inhibition of Autophagy Induced by Atorvastatin Improves Atorvastatin-Induced Apoptosis in J82 Bladder Cancer Cells

**Figure 4.**
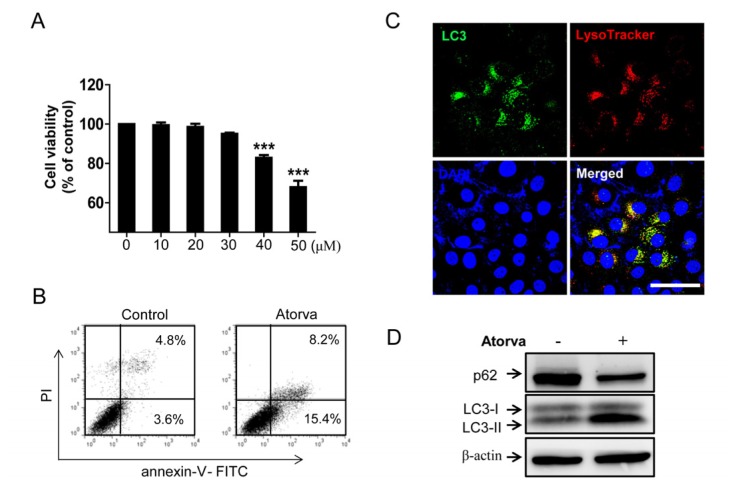

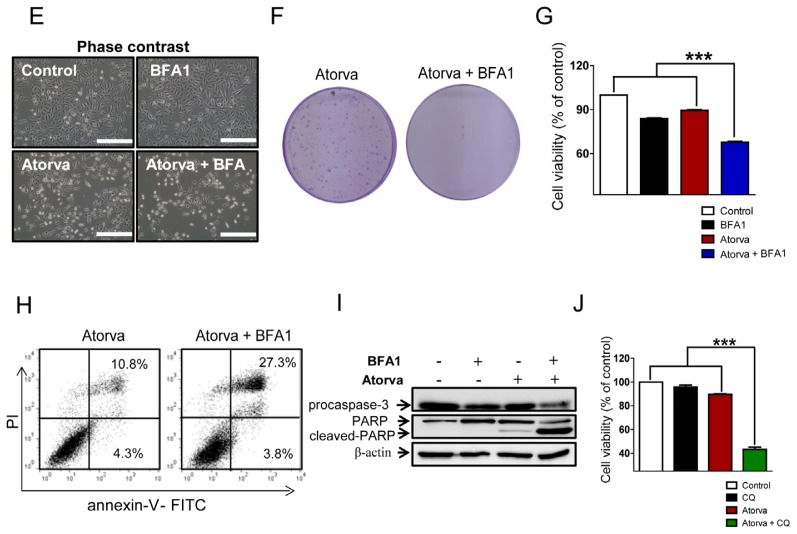
Inhibition of autophagy induced by atorvastatin improves atorvastatin-induced apoptosis in J82 bladder cancer cells. (**A**) The cell viability assay to examine the cytotoxic effects of atorvastatin in J82 cells. Differing concentrations of atorvastatin (zero, 10, 20, 30, 40 and 50 μM) were administrated over 48 h. The values of cell viability are represented by the mean percent of control ± SEM (*n* = 3, *** *p* < 0.001); (**B**) Evaluation of apoptotic cell death after treatments with 30 μM of atorvastatin by flow cytometry analysis with FITC-conjugated annexin-V and PI staining. Relative proportions of both early and late apoptosis are indicated in right lower and right upper quadrant, respectively in each treatment group; (**C**) Immunocytochemistry for the co-localization of LC3 puncta (green) and Lysotracker (red, demarcation for the lysosome) in J82 cells after atorvastatin treatments (30 μM) for 24 h. DAPI was used for nucleus staining. Scale bar = 100 μm; (**D**) Western blot analysis of autophagosome formation markers p62/SQSTM1, LC3-I and LC3-II in untreated (control) and atorvastatin (30 μM) treated J82 cells; (**E**) Phase contrast images of untreated J82 cells (control), treated J82 cells with 20 μM of bafilomycin A1 (BFA1), 20 μM of atorvastatin (Atorva) and both these agents (Atorva + BFA1). Scale bar = 200 μm; (**F**) The clonogenic assay to compare the *in vitro* cell survival potential after treatment with 20 μM of atorvastatin and combined treatment with 20 μM of atorvastatin and BFA1 for 12 days. The colony is defined as containing at least 50 individual cells. Photographs represent each experimental group stained with the clonogenic assay kit; (**G**) The cell viability assay to examine the cytotoxic effects of 20 μM of BFA1, 20 μM of atorvastatin and both these agents. The values of cell viability are represented by the mean percent of control ± SEM (*n* = 3, *** *p* < 0.001); (**H**) Flow cytometry analysis with FITC-conjugated annexin-V and PI staining to evaluate apoptotic cell death in J82 cells treated with 20 μM of atorvastatin and both 20 μM of BFA1 and atorvastatin for 48 h. Relative proportions of both early and late apoptosis are indicated in right lower and right upper quadrant, respectively in each treatment group; (**I**) Western blot analysis of apoptotic markers procaspase-3, total PARP and cleaved-PARP in J82 cells treated with 20 μM of BFA1, 20 μM of atorvastatin and both these agents for 48 h; (**J**) Cell viability assay to compare the cytotoxic effects in J82 cells treated with 20 μM of CQ, 20 μM of atorvastatin and both agents. The values of cell viability are represented by the mean percent of control ± SEM (*n* = 3, *** *p* < 0.001).

Similar to T24 human bladder cancer cells, the cytotoxicity of atorvastatin was observed in J82 human bladder cancer cells in a dose-dependent manner (Figure 4A). In flow cytometry analysis, apoptotic cell death increased after treatment with 30 μM of atorvastatin compared to control (Figure 4B). We found that atorvastatin induced the conspicuous formation of LC3 puncta and Lysotracker co-expressed cells by immunocytochemistry (Figure 4C). Western blot analysis also showed that the conversion of LC3-I into LC3-II increased, while the protein level of p62/SQSTM1 decreased (Figure 4D). Furthermore, combined treatment with atorvastatin and BFA1 strongly enhanced cell death in J82, shown in the phase contrast (Figure 4E), and resulted in the dramatic reduction of the clonogenic capacity of J82 cells compared to atorvastatin-only treated cells (Figure 4F). In addition, cell viability was significantly reduced after combined treatment with atorvastatin and BFA1 when compared to single agent addition (Figure 4G). In flow cytometry analysis, combined atorvastatin and BFA1 treatment group resulted in a higher proportion of apoptotic cell death (Figure 4H). Western blot analysis also showed that combined treatment with atorvastatin and BFA1 increased the expression of cleaved-PARP, while decreasing procaspase-3 and total PARP (Figure 4I). To determine whether other autophagy inhibitor enhance the effects of atorvastatin, CQ was used in J82 cells. As shown in Figure 4J, cell viability was significantly reduced after combined treatment with atorvastatin and CQ when compared to single agent addition. These results suggest that pharmacologic inhibition of autophagy triggered by atorvastatin also potentiates the cytotoxic effects of atorvastatin in J82 human bladder cancer cells by enhancing apoptotic cell death.

### 2.2. Discussion

In this study, we provide evidence that pharmacologic inhibition of autophagy potentiates atorvastatin-induced apoptotic cell death in human bladder cancer cells *in vitro*. Statins have pleiotropic functions, including cholesterol and atherosclerotic plaque reduction, anti-inflammation and anti-cancer effects [15]. Although their potential role in cancer therapy has been researched over the decades, the effects of statins on the prevention and treatment of cancer are still unclear. Pre-clinical and clinical results showed that statins might be beneficial for the treatment of melanoma, leukemia, brain tumor and liver cancer [15]. In bladder cancer, clinical efficacy and the feasibility of statins are still open questions. Experimental study demonstrated that atorvastatin reduced cell proliferation and survival through apoptosis in human bladder cancer cells, RT4 and KU7, *in vitro* [13]. Similarly, our results showed that atorvastatin exhibited significant cytotoxic effects in a dose- and time-dependent manner in human bladder cancer cells *in vitro*. Of note, the cytotoxicity of atorvastatin acts by apoptotic cell death, confirmed by evaluating the expression of several apoptotic markers and annexin-V/PI double staining.

In contrast to the promising results *in vitro*, clinical data have not been hopeful in bladder cancer therapy with statins. Hoffmann *et al*. suggested that statin therapy was significantly associated with tumor progression in non-muscle-invasive bladder cancer (NMIBC) patients treated with intravesical Bacillus Calmette-Guérin (BCG) [10]. A recent retrospective study noted that statins did not improve clinical outcomes, especially in NMIBC patients treated with intravesical BCG immunotherapy [11]. We hypothesized that potential enhancements of the anti-cancer activities of statins can provide a great opportunity to improve the clinical efficacy of statins in bladder cancer therapy.

The role of autophagy activity on the survival of cancer cells is context-dependent as a tumor-suppressing or pro-survival mechanism [24]. Autophagy activation by anti-cancer agents, including tamoxifen, rapamycin, histone deacetylase inhibitors, vitamin D analogues and statins, can result in autophagic cell death, a possible mechanism of tumor cell killing by anti-cancer drugs [18,23]. However, it is controversial whether autophagy acts as a cell death mechanism in the conditions that activate autophagy [25]. In a seeming paradox, autophagy can promote tumor survival when the tumor cells are struggling to endure harsh conditions and, thus, potentially limit the efficacy of anti-cancer drugs [18]. Therefore, targeting the activated autophagy pathway can be a valuable strategy to treat cancer cells combined with anti-cancer agents. Accumulating evidence shows that autophagy inhibition combined with anti-cancer agents can be a novel and effective therapy to treat various human malignancies [22]. The autophagy inhibitor, chloroquine, sensitized colon cancer cells to oxaliplatin under hypoxic conditions *in vitro* and reduced the tumor growth of colon cancer xenografts in bevacizumab- and oxaliplatin-treated mice [26]. Dual inhibition of the PI3K/AKT/mTOR pathway and autophagy activity enhanced the apoptotic cell death of human melanoma cells *in vitro* and *in vivo*, because autophagy activation by mTOR inhibitors may act as a pro-survival mechanism in melanoma cells [27]. In addition, statins activated autophagy via the AMPK signaling pathway in human hepatocellular carcinoma and colorectal carcinoma cells and combined treatments of statins and autophagy inhibitors enhanced cytotoxicity and apoptosis *in vitro* and *in vivo* [28].

In urologic cancers, concurrent inhibition of autophagy during treatment with anti-cancer drugs can improve their anti-cancer efficacy. Autophagy protected Src family kinase (SFK) inhibitor-mediated cell death in human prostate cancer cells, and autophagy inhibition significantly enhanced the tumor cell killing effects of SFK inhibitors *in vitro* [29]. The combination of SFK inhibitors with chloroquine significantly reduced the tumor growth of prostate cancer xenograft in mice [29]. In human renal cell carcinoma cells, autophagy showed resistant activity against mTOR inhibitors; therefore, dual inhibition of mTOR and autophagic pathways effectively induced necroptosis [30]. In this study, atorvastatin triggered autophagy in T24 and J82 human bladder cancer cells. We speculated that autophagy activation attenuates the cytotoxic effects of atorvastatin by its survival mechanism in harsh conditions. As expected, combined treatments with atorvastatin and autophagy inhibitors (BFA1 and CQ) produced higher apoptotic cell death compared with atorvastatin treatment alone in human bladder cancer cells *in vitro*. Our results provide the molecular and cellular clues that combined treatment with autophagy inhibitors can improve statin-induced apoptotic cell death in human bladder cancer cells.

There are several limitations in the present study. We used only pharmacologic inhibitors of autophagy to explore the role of autophagy in atorvastatin-mediated apoptotic cell death. Pharmacologic inhibitors of autophagy are classified as early- and late-stage drugs according to their mechanisms on the pathway of autophagy formation [31,32]. In this study, we used BFA1 (bafilomycin A1) and CQ (chloroquine) as late stage inhibitors. They interfere with lysosomal function and block the lysosomal degradation of autophagosomes [23]. Despite their wide usages, these drugs may result in cytoplasmic accumulation of abnormal autophagosomes, which can be toxic to cells [32]. Thus, we should also examine other chemicals to inhibit autophagy activity, such as 3-methyladenine and verteporfin as early stage inhibitors. To exclude the off-target pharmacologic effects of BFA1 and CQ as autophagy inhibitors, the knockdown of endogenous ATG3, ATG5 or ATG7 expression, which are specifically required for autophagosome formation [33], would be considered by using a genetic approach, such as RNA interference techniques. Such additional experiments offer more convincing evidence that autophagy exerts a pro-survival function in human bladder cancer cells. More importantly, because of the presence of unknown factors that promote the survival of cancer cells in their microenvironment, the therapeutic efficacy of autophagy inhibition *in vitro* and *in vivo* would be different [34]. Therefore, an *in vivo* efficacy study, such as human bladder cancer xenograft model, would be required to support our conclusion that the inhibition of autophagy triggered by atorvastatin provides a potential therapeutic approach to treat bladder cancer.

## 3. Experimental Section

### 3.1. Cell Lines and Culture

Human bladder cancer cell lines (T24 and J82) were purchased from American Type Culture Collection (ATCC, Rockville, MD, USA). T24 and J82 were maintained in Dulbecco’s Modified Eagle’s Medium (DMEM) (WELGENE, Daegu, Korea) supplemented with 20% fetal bovine serum (FBS; WELGENE), 1% penicillin-streptomycin (PenStrep, Invitrogen, Carlsbad, CA, USA) and 1% nonessential amino acids (NEAA; Invitrogen). Low passage numbers of T24 and J82 cells were used for the experiments.

### 3.2. Reagents

Atorvastatin, mevalonate, bafilomycin A1 and rapamycin (Sigma-Aldrich, St. Louis, MO, USA) were dissolved in Dimethyl sulfoxide (DMSO) (Sigma-Aldrich) at an appropriate stock concentration. The following primary antibodies were used in this study: rabbit anti-procaspase-3 antibody (1:1000, Cell Signaling Technology, Danvers, MA, USA), rabbit anti-PARP (1:1000, Cell Signaling Technology), rabbit anti-LC3 (1:2000 for western blot and 1:400 for immunocytochemistry, Novus biological, Littleton, CO, USA), mouse anti-p62 (1:2000, Becton, Dickinson and Company (BD) biosciences, San Jose, CA, USA) and Horseradish Peroxidase (HRP)-conjugated actin (1:2000, Santa Cruz Biotechnology, Dallas, TX, USA).

### 3.3. Cell Viability Assay

Cell viability was determined by the EZ-Cytox cell viability assay kit (iTSBiO, Seoul, Korea). One day before analysis, T24 and J82 were plated in triplicate on a 96-well plate at 2 × 10^4^/well density in a final volume of 100 μL/well DMEM medium and were incubated for 24 h in a 5% CO_2_ environment at 37 °C. Atorvastatin was added at varying concentrations into each well, and human bladder cells were incubated for 24, 48 and 72 h at 37 °C and 5% CO_2_. Ten-microliters of EZ-Cytox Kit reagent were added into each well for 2 h in the standard culture conditions. 96-well plates were gently shaken thoroughly for 5 min on a rocker at room temperature (RT). The absorbance at 450 nm of the treated and untreated samples was measured on a multi-well microplate reader (PerkinElmer, Waltham, MA, USA). DMEM medium supplemented with the same volume of kit reagent on empty wells was used as a blank. Cell viability was represented by percentage values compared to a control.

### 3.4. Immunocytochemistry

To visualize acidic autophagic vacuoles in the cytoplasm, cells were stained with 50 nM of LysoTracker-Red DND-99 fluorescence dye (Life technologies, Carlsbad, CA, USA) for 30 min at RT. Cells were washed with PBS and fixed with 4% formaldehyde (Sigma-Aldrich) for 30 min at RT. After washing three times with PBS containing 0.1% Tween-20 (PBST), cells were permeabilized by treatment with 0.3% Triton X-100 (Sigma-Aldrich) for 20 min. Samples were blocked with 5% donkey serum (Jackson ImmunoResearch Laboratories, West Grove, PA, USA) for one hour at RT with gentle rocking. After blocking, primary antibodies were bound at 4 °C overnight. Samples were rinsed three times with PBST and incubated with secondary antibody for one hour at RT. Samples were washed four times with PBST and incubated in VECTASHIELD™ Mounting Medium containing DAPI (1:1000, Vector Laboratories, Burlingame, CA, USA) for 5 min, followed by additional rinsing twice with PBST. Samples were then examined on a Zeiss LSM 510 confocal microscope (Carl Zeiss Microscopy, Oberkochen, Germany).

### 3.5. Western Blot Analysis

Total cell lysates were prepared in EBC lysis buffer (50 mM Tris-HCl pH 8.0, 300 mM NaCl, 0.5% NP40) containing a proteinase inhibitor cocktail (100 μg/mL lysozyme, 10 μg/mL aprotinin and 10 μg/mL leupeptin, Sigma-Aldrich). The protein concentration was measured by the Bradford protein assay. All samples containing 15 to 20 μg of protein were prepared with 1× Sodium dodecyl sulfate (SDS) loading buffer (60 mM Tris-HCl pH 6.8, 25% glycerol, 2% SDS, 14.4 mM β-mercaptoethanol, 0.1% bromophenol blue). Proteins were separated on 8%, 10% or 12% SDS-PAGE gels, depending on the molecular weight of the target proteins. The gels were then transferred to the nitrocellulose membrane. After one hour of blocking with 5% of skim milk in 1× Tris-Buffered Saline and Tween 20 (TBST), the transferred membranes were blotted with primary antibodies at 4 °C overnight. Membranes were washed three times and incubated with horseradish peroxidase-conjugated secondary antibodies for 45 min. Membranes were washed six times and reacted with Ehanced chemoluminescence (ECL)-chemiluminescent substrate for three min. Blotting images were visualized with a LAS-4000 Charge-Coupled Device (CCD) camera system (Fujifilm, Tokyo, Japan).

### 3.6. Flow Cytometric Analysis

Apoptosis was analyzed by flow cytometry using propidium iodide (PI) and the annexin-V-FITC staining kit (BD Biosciences). Cells were detached by Acutase (Innovative Cell Technologies, San Diego, CA, USA) treatment at 37 °C for 5 min. Detached cells were rinsed twice with DMEM medium and cold PBS by centrifugation of 300× *g* for 5 min. Cells were re-suspended in 1× binding buffer at a concentration of 1 × 10^6^ cells/mL. One hundred microliters of cell buffer solution (1 × 10^5^ cells) was transferred to a round bottom tube and incubated with 5 μL of PI and annexin V-FITC for 15 min at RT in the dark. Finally, 400 μL of 1× binding buffer was added to each sample tube and evaluated by a BD FACSCalibur cytometer (BD Biosciences) according to the company’s instructions. Data were analyzed by using FlowJo software version 7.2.5 (Tree Star Inc., Ashland, OR, USA).

### 3.7. Clonogenic (Colony Formation) Assay

The clonogenic assay was performed to evaluate *in vitro* cell survival after treatment with atorvastatin and autophagy inhibitors. Cells were seeded in 60 mm^2^ (1 × 10^3^/well) and were treated with atorvastatin or both atorvastatin and bafilomycin A1 after 4 h of seeding. Un-treated cells were the negative control of this analysis. All experimental samples were incubated in a 5% CO_2_ environment at 37 °C for 9 to 14 days to form appropriately large clones consisting of at least 50 cells. The clonogenic assay kit (BioPioneer, San Diego, CA, USA) was used to stain colonies. The medium was removed from each of the plates, and samples were fixed with 1 mL of fixing solution for 15 min at RT. Samples were then stained with 1 mL of staining solution for 45 min and washed with PBS three times. Colonies that contained more than 50 cells were observed with a stereomicroscope (Olympus, Shinjuku, Tokyo, Japan).

### 3.8. Statistical Analysis

For statistical analysis, all experiments were performed three times independently with technical duplication in each experiment. All data are represented with the mean and SEM. The statistical significance of the data was evaluated by using either the Student’s *t*-test or one-way analysis of variance (ANOVA) with a post-hoc test of multiple comparisons. Null hypotheses of no difference were rejected if *p*-values were less than 0.05. We performed all statistical analysis using GraphPad Prism 5.0 (GraphPad Software, Inc., La Jolla, CA, USA).

## 4. Conclusions

In summary, our results showed that atorvastatin had pro-apoptotic and autophagy induction activities in human bladder cancer cells *in vitro*. Moreover, combined treatment with autophagy inhibitors improved the cytotoxic effects of atorvastatin by promoting apoptotic cell death. The present study provides new insights into alternative therapeutic approaches for bladder cancer therapy using statins and autophagy inhibition concurrently.

## Supplementary Information



## References

[1] Parkin D.M. (2008). The global burden of urinary bladder cancer. Scandinavian journal of urology and nephrology. Scand. J. Urol. Nephrol.

[2] Shariat S.F., Karam J.A., Lotan Y., Karakiewizc P.I. (2008). Critical evaluation of urinary markers for bladder cancer detection and monitoring. Rev. Urol.

[3] Van Rhijn B.W., Burger M., Lotan Y., Solsona E., Stief C.G., Sylvester R.J., Witjes J.A., Zlotta A.R. (2009). Recurrence and progression of disease in non-muscle-invasive bladder cancer: from epidemiology to treatment strategy. Eur. Urol.

[4] Bellmunt J., Orsola A., Wiegel T., Guix M., de Santis M., Kataja V. (2011). Bladder cancer: ESMO clinical practice guidelines for diagnosis, treatment and follow-up. Ann. Oncol.

[5] Stenzl A., Cowan N.C., de Santis M., Kuczyk M.A., Merseburger A.S., Ribal M.J., Sherif A., Witjes J.A. (2011). Treatment of muscle-invasive and metastatic bladder cancer: Update of the EAU guidelines. Eur. Urol.

[6] Auer J., Berent R., Weber T., Eber B. (2002). Clinical significance of pleiotropic effects of statins: Lipid reduction and beyond. Curr. Med. Chem.

[7] Osmak M. (2012). Statins and cancer: Current and future prospects. Cancer Lett.

[8] Karp I., Behlouli H., Lelorier J., Pilote L. (2008). Statins and cancer risk. Am. J. Med.

[9] Vinogradova Y., Coupland C., Hippisley-Cox J. (2011). Exposure to statins and risk of common cancers: A series of nested case-control studies. BMC Cancer.

[10] Hoffmann P., Roumeguere T., Schulman C., van Velthoven R. (2006). Use of statins and outcome of BCG treatment for bladder cancer. N. Engl. J. Med.

[11] Crivelli J.J., Xylinas E., Kluth L.A., da Silva R.D., Chrystal J., Novara G., Karakiewicz P.I., David S.G., Scherr D.S., Lotan Y. (2013). Effect of statin use on outcomes of non-muscle-invasive bladder cancer. BJU Int.

[12] Da Silva R.D., Xylinas E., Kluth L., Crivelli J.J., Chrystal J., Chade D., Guglielmetti G.B., Pycha A., Lotan Y., Karakiewicz P.I. (2013). Impact of statin use on oncologic outcomes in patients with urothelial carcinoma of the bladder treated with radical cystectomy. J. Urol.

[13] Kamat A.M., Nelkin G.M. (2005). Atorvastatin: A potential chemopreventive agent in bladder cancer. Urology.

[14] Bil J., Zapala L., Nowis D., Jakobisiak M., Golab J. (2010). Statins potentiate cytostatic/cytotoxic activity of sorafenib but not sunitinib against tumor cell lines *in vitro*. Cancer Lett..

[15] Hindler K., Cleeland C.S., Rivera E., Collard C.D. (2006). The role of statins in cancer therapy. Oncologist.

[16] Mizushima N., Komatsu M. (2011). Autophagy: Renovation of cells and tissues. Cell.

[17] Choi A.M., Ryter S.W., Levine B. (2013). Autophagy in human health and disease. N. Engl. J. Med.

[18] Hippert M.M., O’Toole P.S., Thorburn A. (2006). Autophagy in cancer: Good, bad, or both?. Cancer Res.

[19] White E., DiPaola R.S. (2009). The double-edged sword of autophagy modulation in cancer. Clin. Cancer Res.

[20] Guo J.Y., Chen H.Y., Mathew R., Fan J., Strohecker A.M., Karsli-Uzunbas G., Kamphorst J.J., Chen G., Lemons J.M., Karantza V. (2011). Activated Ras requires autophagy to maintain oxidative metabolism and tumorigenesis. Genes Dev.

[21] Zhang J., Yang Z., Xie L., Xu L., Xu D., Liu X. (2013). Statins, autophagy and cancer metastasis. Int. J. Biochem. Cell Biol.

[22] Liu B., Wen X., Cheng Y. (2013). Survival or death: Disequilibrating the oncogenic and tumor suppressive autophagy in cancer. Cell Death Dis.

[23] Yang Z.N.J., Chee C.E., Huang S.B., Sinicrope F.A. (2011). The role of autophagy in cancer: Therapeutic implications. Mol. Cancer Ther.

[24] Wu W.K.K., Coffelt S.B., Cho C.H., Wang X.J., Lee C.W., Chan F.K.L., Yu J., Sung J.J.Y. (2012). The autophagic paradox in cancer therapy. Oncogene.

[25] Jones R.G. (2009). The roles, mechanisms, and controversies of autophagy in mammalian biology. F1000 Biol. Rep.

[26] Selvakumaran M., Amaravadi R.K., Vasilevskaya I.A., O’Dwyer P.J. (2013). Autophagy inhibition sensitizes colon cancer cells to antiangiogenic and cytotoxic therapy. Clin. Cancer Res.

[27] Xie X., White E.P., Mehnert J.M. (2013). Coordinate autophagy and mTOR pathway inhibition enhances cell death in melanoma. PLoS One.

[28] Yang P.M., Liu Y.L., Lin Y.C., Shun C.T., Wu M.S., Chen C.C. (2010). Inhibition of autophagy enhances anticancer effects of atorvastatin in digestive malignancies. Cancer Res.

[29] Wu Z., Chang P.C., Yang J.C., Chu C.Y., Wang L.Y., Chen N.T., Ma A.H., Desai S.J., Lo S.H., Evans C.P. (2010). Autophagy blockade sensitizes prostate cancer cells towards src family kinase inhibitors. Genes Cancer.

[30] Bray K., Mathew R., Lau A., Kamphorst J.J., Fan J., Chen J., Chen H.Y., Ghavami A., Stein M., DiPaola R.S. (2012). Autophagy suppresses RIP kinase-dependent necrosis enabling survival to mTOR inhibition. PLoS One.

[31] Wu Y.Y., Wang X., Guo H.J., Zhang B., Zhang X.B., Shi Z.J., Yu L. (2013). Synthesis and screening of 3-MA derivatives for autophagy inhibitors. Autophagy.

[32] Donohue E., Tovey A., Vogl A.W., Arns S., Sternberg E., Young R.N., Roberge M. (2011). Inhibition of autophagosome formation by the benzoporphyrin derivative verteporfin. J. Biol. Chem.

[33] He C.C., Klionsky D.J. (2009). Regulation mechanisms and signaling pathways of autophagy. Annu. Rev. Genet.

[34] Lu Z., Luo R.Z., Lu Y.L., Zhang X.H., Yu Q.H., Khare S., Kondo S., Kondo Y., Yu Y.H., Mills G.B. (2008). The tumor suppressor gene ARHI regulates autophagy and tumor dormancy in human ovarian cancer cells. J. Clin. Investig.

