# Supplementary material for: Inhibition of Autophagy Potentiates Atorvastatin-Induced Apoptotic Cell Death in Human Bladder Cancer Cells in Vitro
Source: Int J Mol Sci. 2014 May 8;15(5):8106–21. doi: 10.3390/ijms15058106 (PMC4057722; doi:10.3390/ijms15058106)
Supplement: Supplementary file 1 [file ijms-15-08106-s001.pdf]

Supplementary Information

Figure S1. Western blot analysis of autophagy marker LC3-I and LC3-II in T24 cells treated with or without atorvastatin (Atorva) and mevalonate. We added 30 μM of atorvastatin and 50 μM of mevalonate into T24 cells for 24 h.

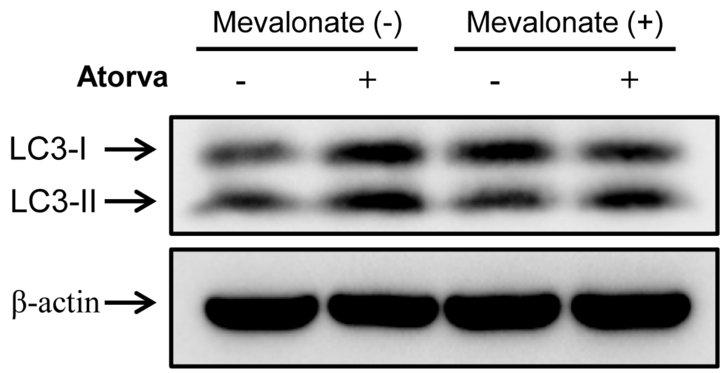

Figure S2. Western blot analysis of autophagy marker LC3-I and LC3-II in T24 cells treated with atorvastatin, bafilomycin A1 (BFA1) and both these agents. All drugs were treated at the concentration of 20 μM for 24 h.

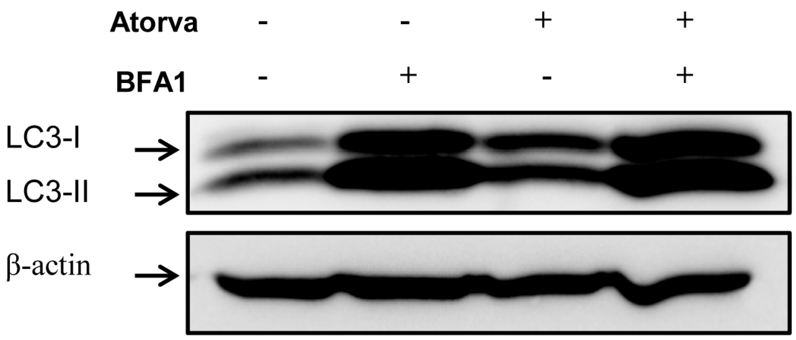

© 2014 by the authors; licensee MDPI, Basel, Switzerland. This article is an open access article distributed under the terms and conditions of the Creative Commons Attribution license (http://creativecommons.org/licenses/by/3.0/).
